# The Most Efficacious Induction Chemotherapy Regimen for Locoregionally Advanced Nasopharyngeal Carcinoma: A Network Meta-Analysis

**DOI:** 10.3389/fonc.2021.626145

**Published:** 2021-02-25

**Authors:** Horace Cheuk-Wai Choi, Sik-Kwan Chan, Ka-On Lam, Sum-Yin Chan, Sze-Chun Chau, Dora Lai-Wan Kwong, To-Wai Leung, Mai-Yee Luk, Anne Wing-Mui Lee, Victor Ho-Fun Lee

**Affiliations:** ^1^ Department of Clinical Oncology, LKS Faculty of Medicine, The University of Hong Kong, Hong Kong, Hong Kong; ^2^ Clinical Oncology Center, The University of Hong Kong-Shenzhen Hospital, Shenzhen, China

**Keywords:** nasopharyngeal carcinoma, induction chemotherapy, survival outcome, network meta-analysis, efficacy

## Abstract

**Background:**

Induction chemotherapy (IC) followed by concurrent chemoradiotherapy (CCRT) for non-metastatic locoregionally advanced nasopharyngeal carcinoma (NPC) has gained considerable attention. However, the most efficacious IC regimens remain investigational. We aimed to compare the survival benefits of all available IC regimens followed by CCRT in this network meta-analysis.

**Methods:**

All randomized-controlled trials of CCRT with or without IC in non-metastatic locoregionally advanced NPC were included, with an overall nine trials of 2,705 patients counted in the analysis. CCRT alone was the reference category. Eight IC regimens followed by CCRT were analyzed: docetaxel + cisplatin (DC), gemcitabine + carboplatin + paclitaxel (GCP), gemcitabine + cisplatin (GP), mitomycin + epirubicin + cisplatin + fluorouracil + leucovorin (MEPFL), cisplatin + epirubicin + paclitaxel (PET), cisplatin + fluorouracil (PF), cisplatin + capecitabine (PX) and cisplatin + fluorouracil (PF), cisplatin + capecitabine (PX). Fixed-effects frequentist network meta-analysis models was applied and P-score was used to rank the treatments.

**Results:**

DC, GP, and PX were the top three IC regimens with the highest probability of benefit on overall survival (OS). Their corresponding hazard ratios (HRs) (95% CIs) compared with CCRT alone were of 0.24 (0.08–0.73), 0.43 (0.24–0.77), and 0.54 (0.27–1.09) and the respective P-scores were 94%, 82%, and 68%. The first three IC regimens showing significantly improved progression-free survival (PFS) were PX, followed by GP and DC with respective HRs of 0.46 (0.24–0.88), 0.51 (0.34–0.77), and 0.49 (0.20–1.20), and P-scores of 82%, 78%, and 74%. Among the studies in the intensity-modulated radiation therapy (IMRT) era, GP and PX were the best performed IC regimens, whilst DC performed the best among non-IMRT studies. Doublet and gemcitabine-based IC regimens had better survival benefits compared to triplet and taxane-based IC regimens, respectively.

**Conclusions:**

Given its consistent superiority in both OS and PFS, DC, GP, and PX ranked among the three most efficacious IC regimens in both the overall and subgroup analysis of IMRT or non-IMRT studies. Exploratory analyses suggested that doublet and gemcitabine-based IC regimens showed better survival performance.

## Introduction

Nasopharyngeal carcinoma (NPC) is endemic in Southern China and Southeast Asia (1). Intensity-modulated radiation therapy (IMRT) with concurrent platinum-based chemotherapy remains the backbone of treatment for non-metastatic locoregionally advanced NPC. Although the locoregional control rate in NPC has been improved, distant metastasis has emerged as the predominant mode of treatment failures. This underlines the potential role for additional systemic therapy (2).

An individual patient data network meta-analysis (NMA) conducted by Meta-Analysis of Chemotherapy in Nasopharynx Carcinoma (MAC-NPC) Collaborative Group demonstrated the potential role of adjunct chemotherapy in the treatment of locoregionally advanced NPC (3). Their study results were further updated recently in the American Society of Clinical Oncology Meeting that induction chemotherapy (IC) followed by concurrent chemoradiotherapy (CCRT) significantly improved distant control and survival from 28 trials with 8214 patients (4). In addition, the recently published phase 3 randomized-controlled trial (RCT) in Hong Kong (NPC-0501) revealed that in contrast to adjuvant chemotherapy, IC particularly using regimen of cisplatin and capecitabine (PX) could potentially improve clinical efficacy (5, 6).

In addition to NPC-0501, other large-scale multicenter phase 3 RCTs have been reported recently. One trial evaluated cisplatin plus gemcitabine (GP) as IC followed by CCRT versus CCRT alone and demonstrated a significantly higher overall survival (OS) and progression-free survival (PFS) (7). Similarly, another trial comparing docetaxel, cisplatin, and fluorouracil (TPF) followed by CCRT showed an improved survival (8). Other trials evaluating IC with different regimens plus CCRT versus CCRT alone were also reported in different period accordingly (9–16). Although IC followed by CCRT has gained considerable attention, the most efficacious regimens for IC remain undefined. To the best of our knowledge, no head-to-head study has yet been conducted that allows for direct comparison of the survival benefits among different IC regimens. We therefore performed this NMA to investigate the differences in survival benefits among all currently available IC regimens followed by CCRT in patients with locoregionally advanced NPC.

## Methods

### Selection Criteria and Search Strategy

To be eligible for this NMA, trials had to evaluate IC plus CCRT versus CCRT alone or to compare different IC regimens. They must be RCTs and include patients with previously untreated non-metastatic locoregionally advanced NPC. Trials were eligible if at least 60 patients had been included (17). Retrospective studies were excluded. Similarly, trials evaluating IC plus CCRT versus adjuvant chemotherapy plus CCRT or adjuvant chemotherapy plus CCRT versus CCRT alone were eligible for sensitivity analysis.

We performed a systematic literature search using PubMed/MEDLINE Ovid, Embase, Cochrane Library, CINAHL Databases, trial registries and other sources, in accordance with the Preferred Reporting Items for Systematic Review and Meta-analysis (PRISMA) guidelines for publications which included IC followed by CCRT in locoregionally advanced NPC (**Supplementary Appendix** and **Supplementary Figure 1**). Only full-length published articles written in English were included.

### Data Extraction

Data extraction was performed by two reviewers (HCWC and SKC). Reported data for any relevant variable for which analysis was conducted were extracted. These included (1) study characteristics including country, year of publication and phase; (2) number of patients in each arm, regimens compared, and treatment protocol; (3) reported hazard ratio (HR) and 95% confidence interval (CI) including OS and PFS.

### End Point Definitions

The primary end point for this NMA was OS, defined as the time from the date of randomization until the date of death from any cause. The secondary end point was PFS, the time from the date of randomization to the date of first disease progression (locoregional or distant) or death from any cause, whichever occurred earlier.

### Quality Assessment

Two authors (HCWC and SKC) scored each included study using the modified Jadad system (18). Randomization (0, 1, or 2), double-blinding (0, 1, or 2), recording of dropouts and/or withdrawals (0 or 1), and allocation concealment (0, 1, or 2) were assessed. A score of 4 or above is indicative of high quality.

### Statistical Analysis

We performed this NMA using a frequentist approach. The I^2^ and Q statistic were used to quantify the heterogeneity among different trials for the same IC regimen (19). Fixed-effects model was used in this study while random-effects model was planned in the case of important heterogeneity if I^2^ > 50% and/or significant Q statistic at *p* < 0.1. The IC regimens were ranked using the P-score where regimens having higher P-score represent better performance (20). Sensitivity analysis of including trials involving adjuvant chemotherapy plus CCRT was also performed.

In view of the importance of radiotherapy (RT) technique in managing NPC, we performed a subgroup analysis stratified according to RT technique (IMRT trials versus non-IMRT trials). We arbitrarily considered trials with >50% patients treated with IMRT as studies which adopted significant use of IMRT, since not all trials clearly depicted the exact number or percentage of patients who received IMRT and there has been so far no universal consensus on this issue. Owing to the relatively small number of studies in this NMA, adjustments for other potential confounders and other stratified analyses addressing potential sources of heterogeneity including study design, sources and study location were not conducted. Publication bias could not be formally evaluated in the NMA because of the small number of studies included. Albeit the real potential for this bias given the relatively small number of studies, we judged the certainty in the evidence was unlikely to be decreased by this concern.

Furthermore, we conducted exploratory analyses to examine the intervention effect in different contexts by comparing doublet and triplet IC regimens, as well as gemcitabine-based and taxane-based regimens (docetaxel or paclitaxel + platinum).

All analyses were conducted using R version 3.6.3 (R Foundation for Statistical Computing, Vienna, Austria) and *p* < 0.05 was considered statistically significant.

## Results

### Quality Assessment of Included Studies

The NMA consisted of 9 trials and 2,705 patients (6–11, 13, 15, 16). Study design and quality assessment are shown (**Supplementary Table 1, 2**). Seven trials recruited patients with stage III to IVB disease based on the 5^th^, 6^th^, and 7^th^ edition of American Joint Committee on Cancer Staging Manual (AJCC-5, AJCC-6, and AJCC-7, respectively) and Union for International Cancer Control (UICC) (1997) (6–9, 11, 13, 16). The HeCoG RCT conducted by Fountzilas et al. also included patients with stage IIB NPC (staged by AJCC-6) accounting for 20.6% (29 out of 141 patients) of the whole study population (10), while the GORTEC 2006-02 RCT conducted by Frikha et al. recruited patients with stage T2b, T3, T4, and/or N1-N3 disease staged by AJCC-7 without further elaboration on the overall stage distribution (15).

**Figure 1:**
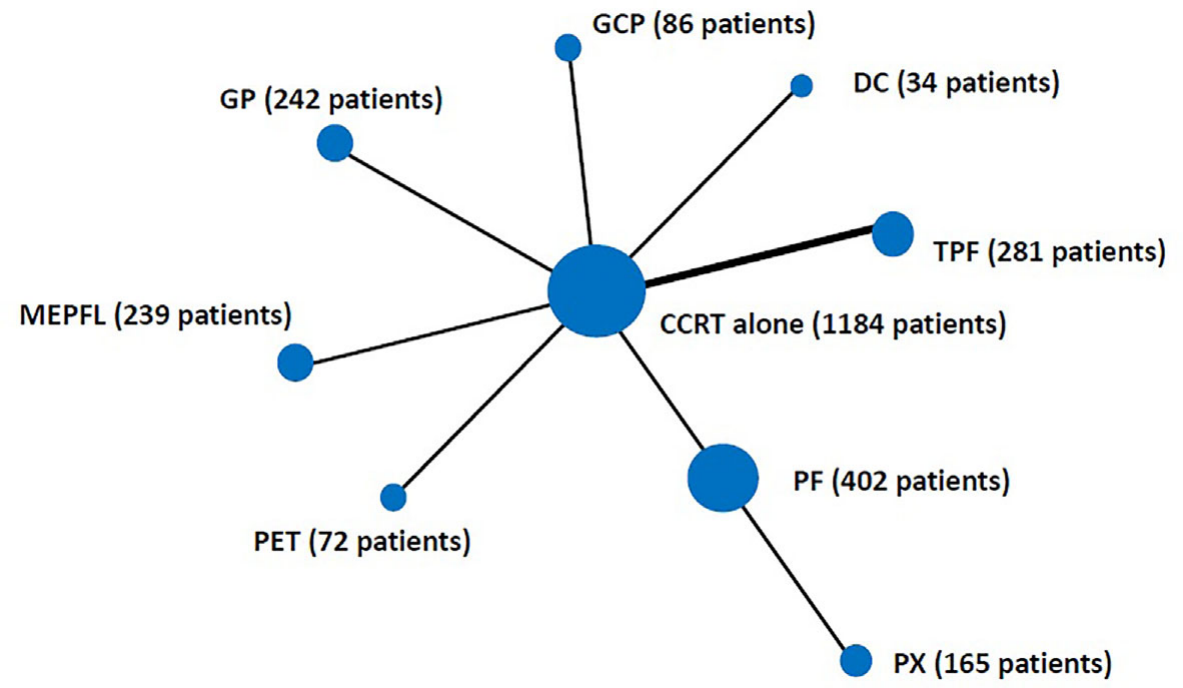
Schematic of the network of evidence used in network meta-analysis for induction chemotherapy. The size of the nodes is proportional to the number of patients in each induction chemotherapy regimen category. The width of the lines is proportional to the number of comparisons. Two trials were included in the comparison of CCRT vs TPF. CCRT, concurrent chemoradiation; DC, docetaxel + cisplatin; PET, cisplatin + epirubicin + paclitaxel; GCP, gemcitabine + carboplatin + paclitaxel; TPF, docetaxel + cisplatin + fluorouracil; MEPFL, mitomycin + epirubicin + cisplatin + fluorouracil + leucovorin; PF, cisplatin + fluorouracil; PX, cisplatin + capecitabine; GP, gemcitabine + cisplatin.

CCRT alone was the reference category. IC regimens followed by CCRT were grouped into eight categories: docetaxel + cisplatin (DC), cisplatin + epirubicin + paclitaxel (PET), gemcitabine + carboplatin + paclitaxel (GCP), TPF, mitomycin + epirubicin + cisplatin + fluorouracil + leucovorin (MEPFL), cisplatin + fluorouracil (PF), cisplatin + capecitabine (PX) and gemcitabine + cisplatin (GP). The network is displayed in **Figure 1**.

### Overall Survival

**Figure 2:**
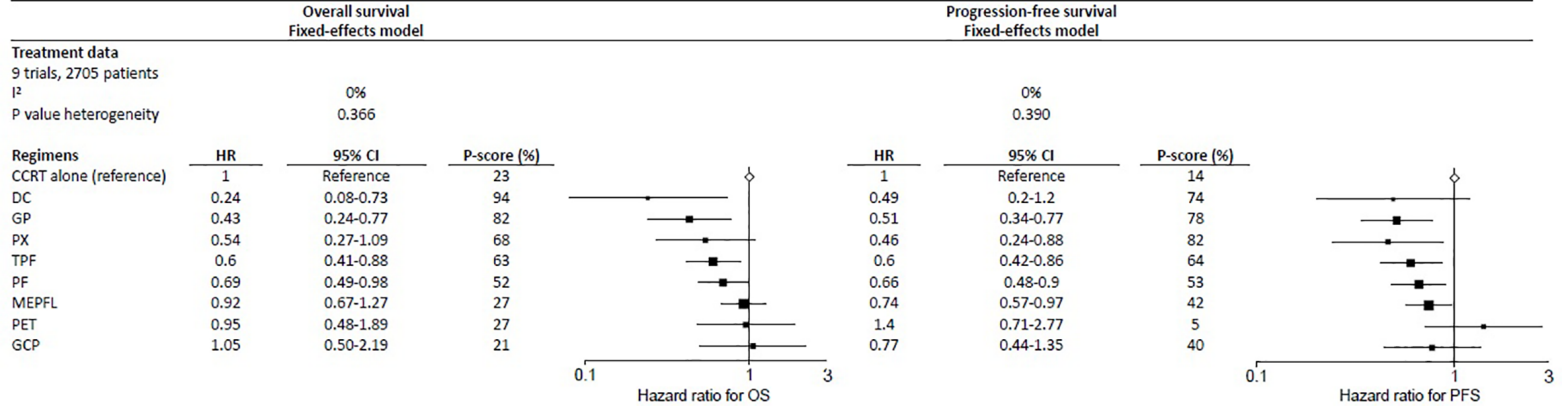
Forest plot for overall survival (left) and progression-free survival (right) showing results comparing IC regimens against CCRT from network meta-analysis. HR<1 is in favor of CCRT alone. 95% CI, 95% confidence interval; HR, hazard ratio; PFS, progression-free survival; OS, overall survival; regimens analyzed: CCRT, concurrent chemoradiation; DC, docetaxel + cisplatin; PET, cisplatin + epirubicin + paclitaxel; GCP, gemcitabine + carboplatin + paclitaxel; IC, induction-concurrent; TPF, docetaxel + cisplatin + fluorouracil; MEPFL, mitomycin + epirubicin + cisplatin + fluorouracil + leucovorin; PF, cisplatin + fluorouracil; PX, cisplatin + capecitabine; GP, gemcitabine + cisplatin.

The three IC regimens that had the highest significant benefit on OS were DC, followed by GP and PX, with respective P-score of 94%, 82%, and 68%, where a higher score meant a higher probability of being the best IC regimen (**Figure 2**). Their corresponding HRs (95% CIs) compared with CCRT alone were 0.24 (0.08–0.73), 0.43 (0.24–0.77), and 0.54 (0.27–1.09). There was no significant heterogeneity observed (I^2^ = 0%; *p* = 0.366 for Q statistic) and fixed-effects model was used.

### Progression-Free Survival

The results for PFS were presented using a fixed-effects model because of no heterogeneity (I^2^ = 0%; *p* = 0.390 for Q statistic) detected. The three best performed regimens in PFS were slightly different from OS, with PX being the most effective, with a P-score of 82%; GP and DC with respective P-scores of 78% and 74%, ranked second and third (**Figure 2**). Their corresponding HRs (95% CIs) were 0.46 (0.24–0.88), 0.51 (0.34–0.77), and 0.49 (0.20–1.20) respectively.

### Sensitivity Analyses

The sensitivity analysis was performed for both OS and PFS after including two trials which involved adjuvant chemotherapy plus CCRT (**Supplementary Figures 2**, **3** and **Supplementary Table 1**, **2**) (6, 21). Three comparisons were added, namely, induction PF plus CCRT versus CCRT followed by adjuvant PF, induction PX plus CCRT versus CCRT followed by adjuvant PF and CCRT followed by adjuvant PF versus CCRT alone. The results of the sensitivity analysis in OS were in agreement with those in initial NMA. DC remained ranked first and GP and PX was ranked second and third (**Supplementary Figure 4**). No heterogeneity (I^2^ = 0%, *p* = 0.610 for Q statistic) was detected in OS. The sensitivity analysis in PFS did not significantly modify network estimates neither; the three first regimens remained the same and there was no significant heterogeneity (I^2^ = 0%, *p* = 0.601 for Q statistic).

### Subgroup Analyses

Subgroup analysis was performed after stratifying the trials into IMRT studies and non-IMRT studies as we defined previously (**Supplementary Table 1**). In the IMRT trials, GP and PX were the best performed IC regimens in OS with respective P-scores of 90% and 75%, respectively. Similar performance was also observed in PFS with their corresponding P-scores of 79% and 84%, respectively. In the non-IMRT trials, DC performed best in both OS and PFS and the P-scores were 99% and 95% respectively (**Supplementary Figure 5**).

### Exploratory Analyses

When compared with CCRT alone, doublet IC regimens had significantly better survival benefits over triplet IC regimens in both OS (HR (95% CI) 0.52 (0.34–0.79) vs 0.73 (0.49–1.10); P-score 94% vs 53%) and PFS (HR (95% CI) 0.58 (0.43–0.79) vs 0.74 (0.54–1.02); P-score 93% vs 55%) (**Supplementary Figure 6**). On the other hand, gemcitabine-based IC regimen had better performance than taxane-based IC regimens in OS (HR (95% CI) 0.43 (0.24–0.77) vs 0.62 (0.45–0.85); P-score 93% vs 57%) and PFS (HR (95% CI) 0.51 (0.34–0.77) vs 0.70 (0.53–0.91); P-score 95% vs 55%) when compared with CCRT alone (**Supplementary Figure 6**).

## Discussion

IC followed by CCRT for previously untreated locoregionally advanced NPC has gained increasing popularity. Several RCTs have investigated the efficacy of IC in addition to CCRT (5–16). Albeit such encouraging results, the most efficacious IC regimen remains undefined. However, it is rather difficult and impractical to conduct a well-designed phase 3, multicenter, RCT directly comparing different IC regimens due to the constraints of resources and a very long event follow-up duration. The current NMA summarizes up-to-date evidence on the efficacy of different IC regimens using quantitative methods. NMA yields summary estimates for the relative effectiveness between different intervention pairs, and ranks them according to the outcomes measured (22). It has been widely applied to studies in NPC and head and neck squamous cell carcinomas and was able to forecast the results of RCTs published afterwards (3, 4, 23–25). To the best of our knowledge, this NMA is the first to evaluate various IC regimens in previously untreated locoregionally advanced NPC.

The major findings of our study can be summarized as follows. First, DC, GP, and PX ranked better than other regimens for OS improvement. Second, when considering PFS, IC regimens of PX, GP, and DC ranked better. These results were robust to sensitivity analyses and subgroup analyses though they were not entirely consistent between two survival end points. While TPF and PF also showed significant improvement in both OS and PFS, they had lower P-scores which make them being ranked in a lower place. Given its consistent superiority in both OS and PFS over the other regimens, DC, GP, and PX could be considered the most efficacious and more preferred IC regimens. Third, in our exploratory analyses, doublet, and gemcitabine-based IC regimens were superior to triplet and taxane-based IC regimens respectively.

The choice of a most suited IC regimen for a given patient should also take changes in quality of life and cost-effectiveness into consideration. A recent study in China demonstrated the cost-effectiveness of induction GP compared to induction TPF for patients with locoregionally advanced NPC (26). On the other hand, the most optimal number of cycles of IC and the most optimal time interval between IC and subsequent radiation therapy are less well defined. Two retrospective studies in China respectively revealed that two cycles of IC were good enough to attain locoregional control and that prolonged interval of more than 30 days between IC and RT was associated with a high risk of distant failure (27, 28). However, these two studies were limited to the regimens of PF, DC, and TPF. Last but not least, future research on biomarker studies during and after IC to identify and predict good and poor responders to IC is urgently warranted. Lv et al. in their retrospective study of 673 patients reported the plasma Epstein-Barr virus (EBV) (deoxyribonucleic acid) DNA clearance kinetics during IC was prognostic of survival (29). In particular, early responders with more rapid clearance of plasma EBV DNA during IC had a significantly longer OS. Similarly, our another recently published prospective study also demonstrated that the half-life clearance rate of plasma EBV DNA of 15 days, which is during the very early phase of radical treatment, was prognostic of distant metastasis-free survival (DMFS), PFS and OS (30). These two studies indicated the potential utility of real-time monitoring of plasma EBV DNA during IC for risk-adapted treatment intensity modification. While we are still investigating and identifying the most effective predictive biomarkers during and after IC, these efforts have paved the way in evaluating the value of plasma EBV DNA in NPC risk stratification for future personalized treatment strategies.

We believe that our NMA represents the most updated study currently with high-quality data and rigorous methodology, which are major strengths of our work. We will certainly include ongoing trials and trials which are just completed in our analysis in the future once their results are available. Nonetheless, there are a few limitations in our study. Due to the lack of comprehensive and homogenous data of acute toxicity, along with few reliable data on late toxicity, it is not possible to compare safety profiles among different IC regimens *via* meta-analysis in this study. Besides, old radiation techniques were employed in some of the studies in this NMA. It would rather difficult to evaluate if radiation techniques would impact on survival outcomes and toxicities given the few trials involved for each IC regimens, even if these data were recorded adequately and appropriately. Albeit such limitation, we tried our best to summarize the major acute toxicities of each IC regimen here (**Supplementary Table 3** and **4**). In addition, we did not include multiple survival end points such as DMFS and locoregional recurrence-free survival for comparison in our network because these end points were not all available and standardized among the studies included. Finally, although NMA is currently well accepted by multiple public health agencies or authorities as one of the strategies to conduct evidence synthesis systematically and the related guidelines have been published for the evaluation of healthcare interventions (31, 32), it should be borne in mind the limitations of NMA associated with the use of such indirect comparisons. Although our study provided IC regimen ranking in OS and PFS which is considered as an attractive output of NMA, one should be reminded that the computation of ranking probabilities relies mainly on the point estimates which is the HRs in this study (20). In order to accurately and critically evaluate the evidence and certainty that an IC regimen is superior to another, instead of paying attention to the individual regimen ranking, more emphasis should be put to the HR estimates and their corresponding CIs, as well as the consistency of their HR estimates across various survival end points.

## Conclusion

In conclusion, DC, GP, and PX were the most efficacious IC regimens for locoregionally advanced NPC in this NMA. Clinical judgment with comprehensive evaluation of risk of recurrence and potential treatment-related toxicities should be carefully exercised in this setting. Additional data and more clinical studies are warranted to help devise personalized treatments that suit individual needs.

## Author’s Note

Part of the results of this manuscript was presented as an abstract in ESMO Congress 2020 on September 19 to 21, 2020.

## Data Availability Statement

The original contributions presented in the study are included in the article/**Supplementary Material**. Further inquiries can be directed to the corresponding author.

## Author Contributions

HCC, SKC, AWL, and VHL designed the study. HCC, SKC, and VHL performed the statistical analysis. All authors contributed patient data and participated in reviewing and improving statistical analysis and manuscript. All authors contributed to the article and approved the submitted version.

## Conflict of Interest

The authors declare that the research was conducted in the absence of any commercial or financial relationships that could be construed as a potential conflict of interest.
