# Supplementary material for: The Most Efficacious Induction Chemotherapy Regimen for Locoregionally Advanced Nasopharyngeal Carcinoma: A Network Meta-Analysis
Source: Front Oncol. 2021 Feb 25;11:626145. doi: 10.3389/fonc.2021.626145 (PMC7951057; doi:10.3389/fonc.2021.626145)
Supplement: Supplementary file 1 [file DataSheet_1.docx]

**Supplementary Materials**

**The Most Efficacious Induction Chemotherapy Regimen for Locoregionally Advanced Nasopharyngeal Carcinoma: A Network Meta-analysis**

Horace Cheuk-Wai Choi, Sik-Kwan Chan, Ka-On Lam, Sum-Yin Chan, Sze-Chun Chau, Dora Lai-Wan Kwong, To-Wai Leung, Mai-Yee Luk, Anne Wing-Mui Lee, and Victor Ho-Fun Lee

**Supplementary Appendix. Methodology of Network Meta-analysis**

**Supplementary Figure 1.** Flowchart showing the results of systematic review identified from PubMed/MEDLINE, Ovid, Embase, Cochrane Library, CINAHL Databases, trial registries and other sources

**Supplementary Figure 2.** Flowchart showing the results of systematic review identified from PubMed/MEDLINE, Ovid, Embase, Cochrane Library, CINAHL Databases, trial registries and other sources, for sensitivity analysis

**Supplementary Figure 3.** Graphical representation of the trial network for overall and progression-free survival in sensitivity analysis

**Supplementary Figure 4.** Summary of network meta-analysis results in sensitivity analysis

**Supplementary Figure 5.** Summary of network meta-analysis results in subgroup analysis

**Supplementary Figure 6.** Summary of network meta-analysis results in exploratory analyses

**Supplementary Table 1.** Details of randomized-controlled trials in the current network meta-analysis and sensitivity analysis

**Supplementary Table 2.** Quality assessment of the randomized-controlled trials selected

**Supplementary Table 3.** Major acute adverse events during induction chemotherapy phase of each trial

**Supplementary Table 4.** Major acute adverse events during concurrent chemoradiotherapy phase of each trial

**Supplementary Appendix. Methodology of network meta-analysis**

We performed a comprehensive systematic literature search, in accordance with the Preferred Reporting Items for Systematic Review and Meta-analysis (PRISMA) guidelines for full-length journal publications which included previously untreated locoregionally advanced non-metastatic NPC treated with induction chemotherapy followed by concurrent chemoradiotherapy. The PubMed/MEDLINE Ovid, Embase, Cochrane Library, CINAHL Databases, trial registries and other sources were employed and searched from 1990 to the present day, with the most recent search carried out on April 30, 2020. Key words and medical sub-heading (MeSH) terms used in the search strategy covered the following concepts: “nasopharyngeal”, “induction”, and “prospective”. We aimed at identifying any journal articles which published the use of induction chemotherapy followed by concurrent chemotherapy and any forms of radiation therapy to the primary tumour and the neck. On the other hand, we also aimed at identifying any journal articles which evaluated concurrent-induction chemoradiotherapy versus concurrent-adjuvant chemoradiotherapy, and concurrent-adjuvant chemoradiotherapy versus concurrent chemoradiotherapy alone, for sensitivity analysis. Key words and MeSH terms used covered the concepts above as well as “adjuvant”. The key words and MeSH terms within each concept were then separated by the Boolean operator “AND”. Only full-length journal articles written in English were included. The details and results of the literature search was provided (supplementary Figure 1 and 2, and supplementary Table 1).

**Supplementary Figure 1.** PRISMA flowchart showing the results of systematic review identified from PubMed/MEDLINE, Ovid, Embase, Cochrane Library, CINAHL Databases, trial registries and other sources.

Records identified from PubMed/MEDLINE, Ovid, Embase, Cochrane Library and CINAHL: nasopharyngeal, induction, and prospective

(N = 124)

Studies included

 (n = 9)

(n = )

Prospective studies

(n = 15)

Records screened

(n = 112)

Studies assessed for eligibility

(n = 41)

Excluded (n = 12)

Duplications

Excluded (n = 71)

Irrelevant comparisons

Excluded (n = 26)

Retrospective studies
Case reports
Absence of concurrent chemoradiation

Excluded (n = 6)

Preliminary results of the studies included
Ongoing trials
Completed trials without results reported

**Supplementary Figure 2.** PRISMA flowchart showing the results of systematic review identified from PubMed/MEDLINE, Ovid, Embase, Cochrane Library, CINAHL Databases, trial registries and other sources, for sensitivity analysis.

Records identified from PubMed/MEDLINE, Ovid, Embase, Cochrane Library and CINAHL: nasopharyngeal, induction, adjuvant, and prospective

(N = 120)

Studies included

 (n = 2)

(n = )

Prospective studies

(n = 5)

Records screened

(n = 114)

Studies assessed for eligibility

(n = 21)

Excluded (n = 6)

Duplications

Excluded (n = 93)

Irrelevant comparisons

Excluded (n = 16)

Retrospective studies
Case reports
Absence of concurrent chemoradiation

Excluded (n = 3)

Preliminary results of the studies included

**Supplementary Figure 3.** Graphical representation of the trial network for overall survival and progression-free survival in sensitivity analysis.


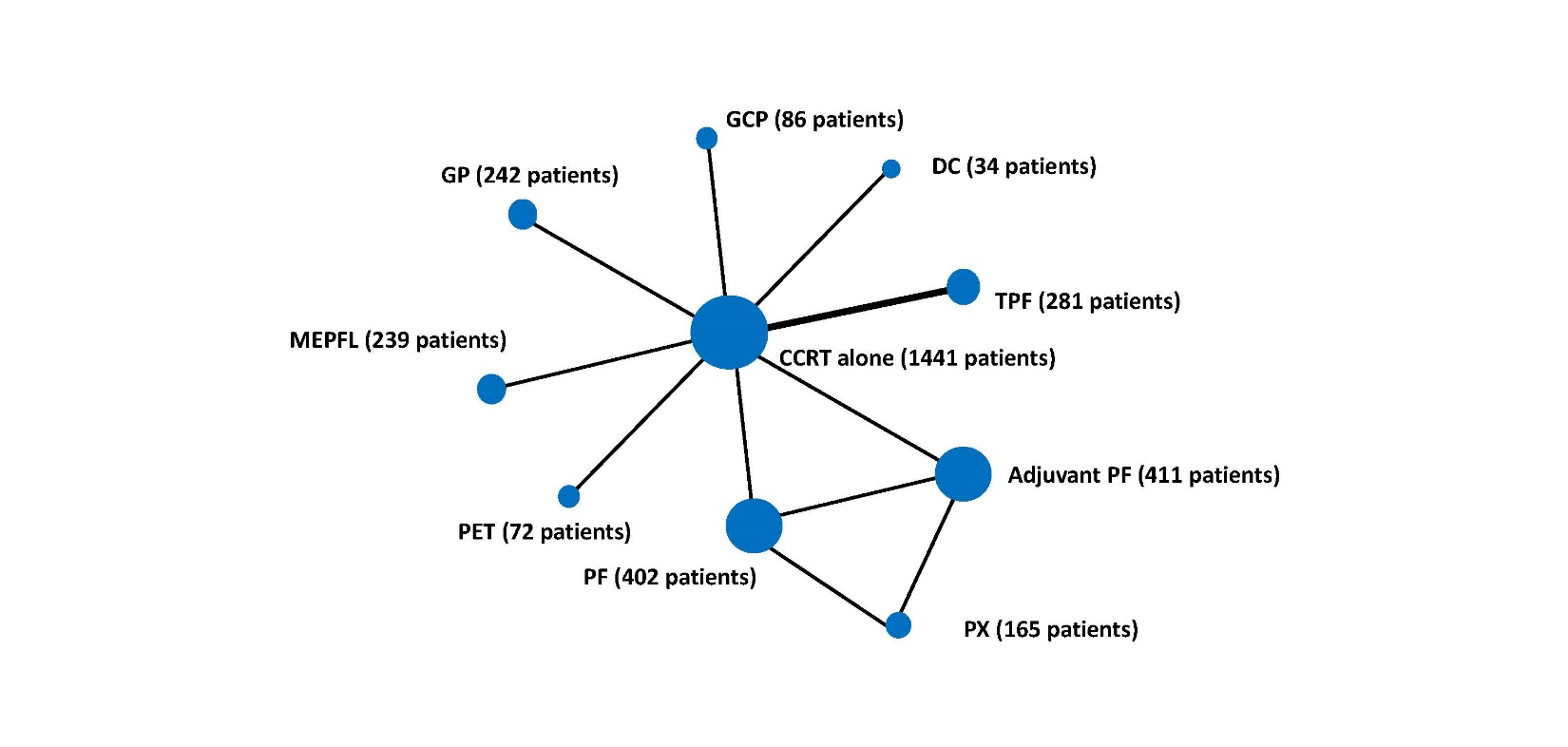


The size of the nodes is proportional to the number of patients in each induction regimen category. The width of the lines is proportional to the number of comparisons. Two trials were included in the comparison of CCRT vs TPF.

CCRT, concurrent chemoradiation; DC, docetaxel + cisplatin; PET, cisplatin + epirubicin + paclitaxel; GCP, gemcitabine + carboplatin + paclitaxel; TPF, docetaxel + cisplatin + fluorouracil; MEPFL, mitomycin + epirubicin + cisplatin + fluorouracil + leucovorin; PF, cisplatin + fluorouracil; PX, cisplatin + capecitabine; and GP, gemcitabine + cisplatin.

**Supplementary Figure 4.** Forest plot for overall survival (left) and progression-free survival (right) showing results comparing IC regimens against CCRT from network meta-analysis under sensitivity analysis.


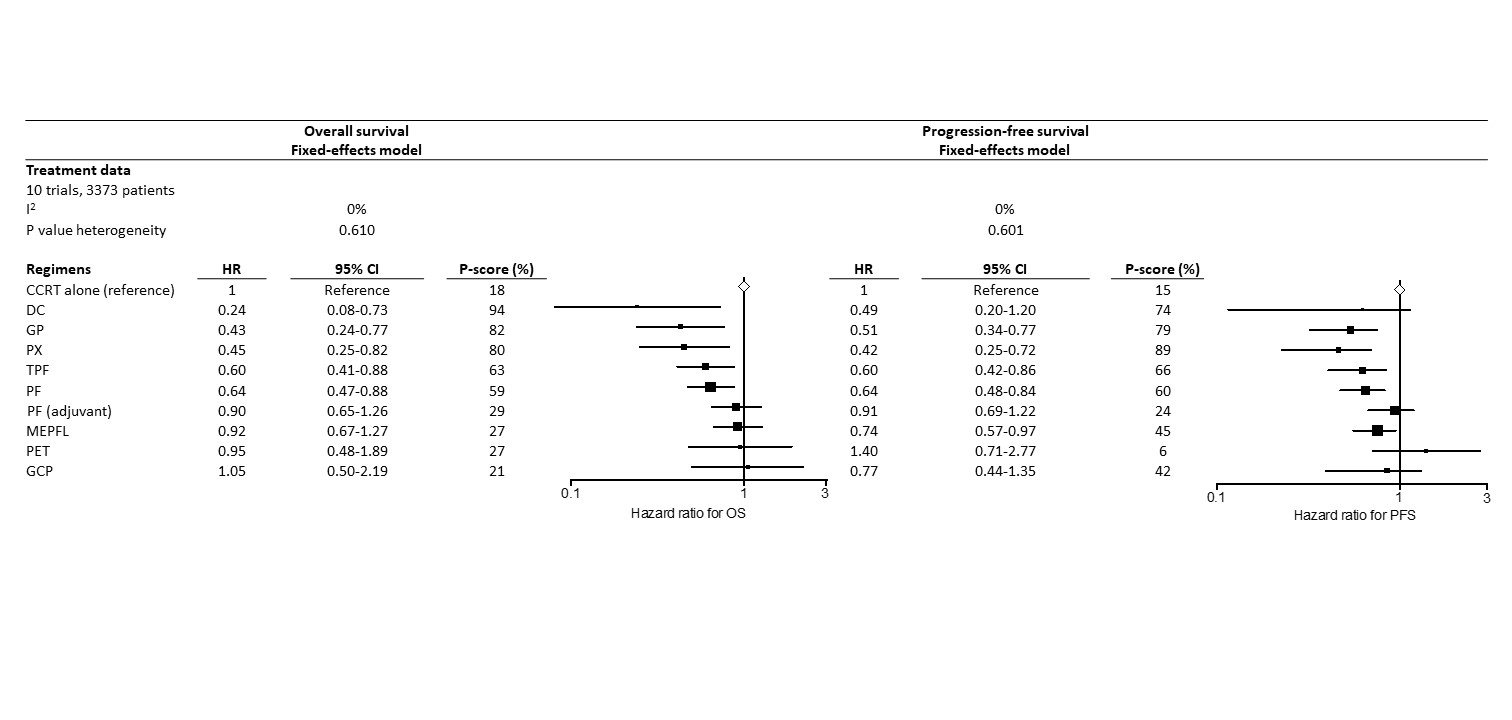


HR<1 is in favor of CCRT alone. 95% CI, 95% confidence interval; HR, hazard ratio; PFS, progression-free survival; OS, overall survival; regimens analyzed: CCRT, concurrent chemoradiation; DC, docetaxel + cisplatin; PET, cisplatin + epirubicin + paclitaxel; GCP, gemcitabine + carboplatin + paclitaxel; TPF, docetaxel + cisplatin + fluorouracil; MEPFL, mitomycin + epirubicin + cisplatin + fluorouracil + leucovorin; PF, cisplatin + fluorouracil; PX, cisplatin + capecitabine; GP, gemcitabine + cisplatin.

**Supplementary Figure 5.** Forest plot for overall survival (left) and progression-free survival (right) showing results comparing IC regimens against CCRT from network meta-analysis in the subgroup analysis stratified by RT technique (IMRT studies (up) and non-IMRT studies(down))


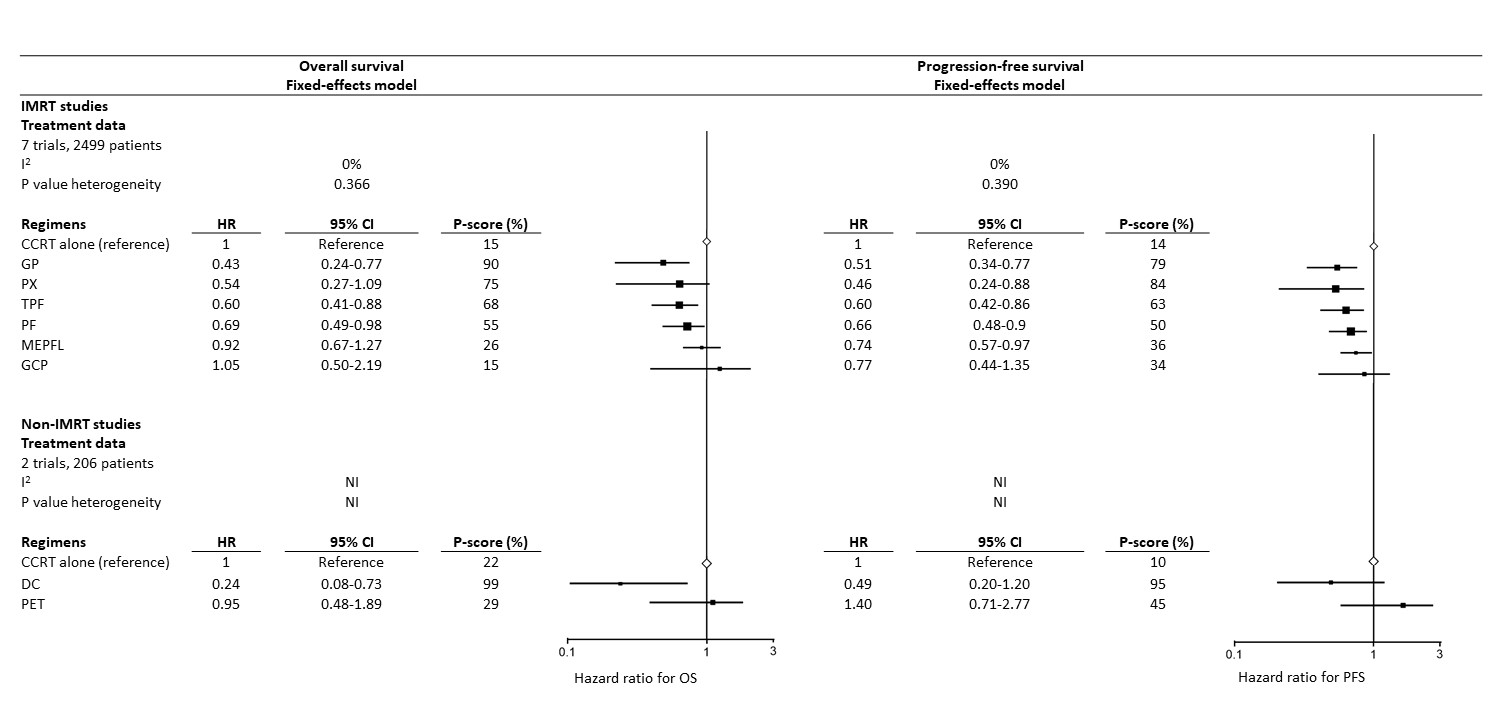


HR<1 is in favor of CCRT alone. 95% CI, 95% confidence interval; HR, hazard ratio; PFS, progression-free survival; OS, overall survival; NI, not inducible; IMRT, intensity-modulated radiation therapy; RT, radiotherapy; regimens analyzed: CCRT, concurrent chemoradiation; DC, docetaxel + cisplatin; PET, cisplatin + epirubicin + paclitaxel; GCP, gemcitabine + carboplatin + paclitaxel; TPF, docetaxel + cisplatin + fluorouracil; MEPFL, mitomycin + epirubicin + cisplatin + fluorouracil + leucovorin; PF, cisplatin + fluorouracil; PX, cisplatin + capecitabine; GP, gemcitabine + cisplatin.

**Supplementary Figure 6.** Forest plot for overall survival (left) and progression-free survival (right) showing results comparing doublet and triplet IC regimens (up) and comparing gemcitabine-based and taxane-based IC regimens against CCRT (down) from network meta-analysis in the exploratory analyses


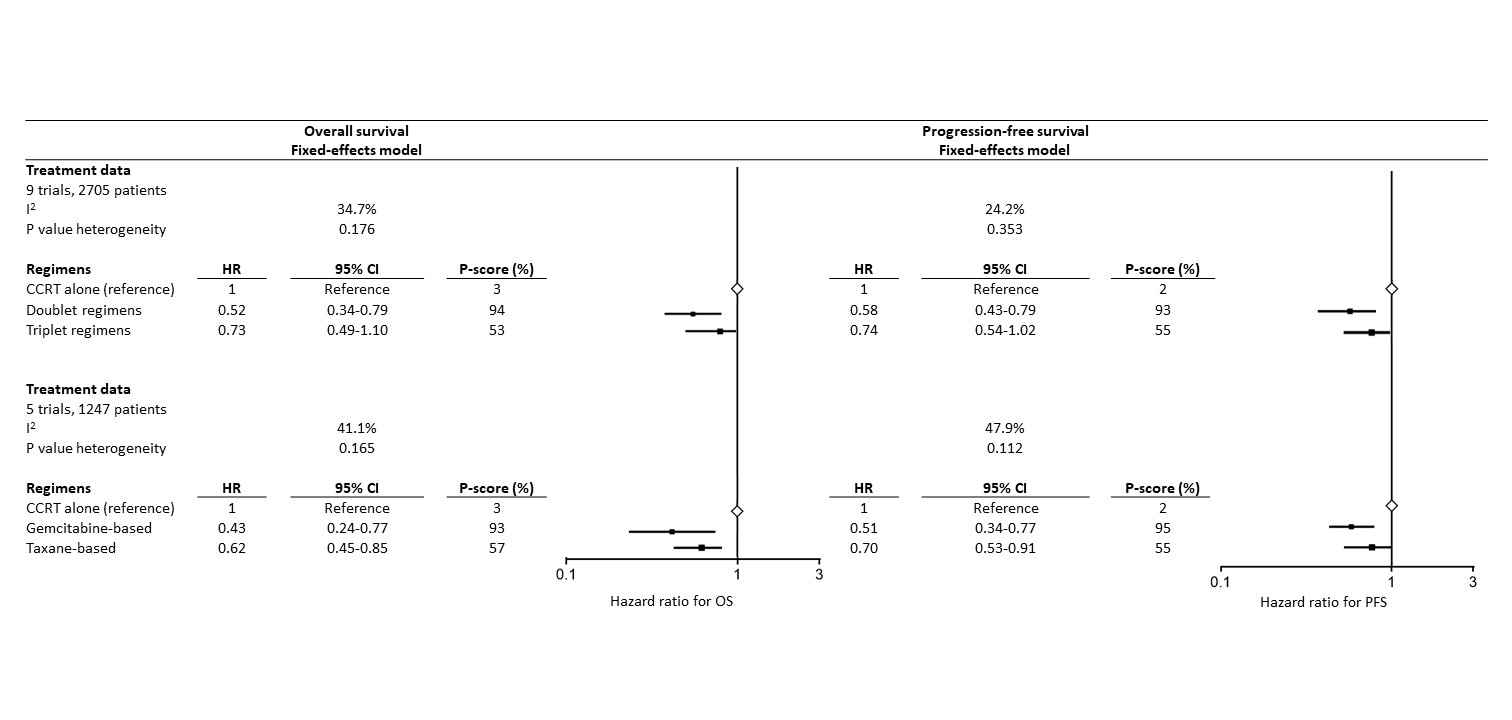


The study of Tan (11) was omitted when comparing gemcitabine-based and taxane-based IC regimens against CCRT because the IC regimen in this study contains both gemcitabine and paclitaxel.

**Supplementary Table 1. Details of randomized-controlled trials in the current network meta-analysis and sensitivity analysis**

| **Trials** | **Phase** | **Sample Size,**  **n** | **Stage Eligibility** | **No. of Patients in Experimental**  **Arm, n** | **No. of Patients in Control Arm, n** | **Experimental Arm of Chemotherapy**  **Regimen** | **Control Arm of Chemotherapy Regimen** | **Significant use of IMRT^a^** | **RT dose** | **OS HR  (95% CI)** | **PFS HR  (95% CI)** |
| --- | --- | --- | --- | --- | --- | --- | --- | --- | --- | --- | --- |
| Hui EP (9) | II | 65 | III-IVB (UICC, 1997) | 34 | 31 | IC: docetaxel 75 mg/m^2^ day 1;  cisplatin 75 mg/m^2^ day 1; Q3W  for 2 cycles  CC: cisplatin 40 mg/m^2^ day 1;  Q1W for 8 cycles | CC: cisplatin 40  mg/ m^2^ day 1;  Q1W for 8 cycles | N | T 66 Gy | 0.24  (0.08–0.73) | 0.49  (0.20–1.19) |
| Fountzilas G (10) | II | 141 | IIB-IVB (AJCC-6) | 72 | 69 | IC: cisplatin 75 mg/ m^2^ day 1;  epirubicin 75 mg/ m^2^ day 1;  paclitaxel 175 mg/ m^2^ day 1;  Q3W for 3 cycles  CC: cisplatin 40 mg/ m^2^ day 1;  Q1W for 8 cycles | CC: cisplatin 40  mg/ m^2^ day 1;  Q1W for 8 cycles | N | T 66-70 Gy/6.5-7 weeks; N-50 Gy; N+ 66-70Gy | 0.95  (0.48–1.89) | 1.40  (0.71–2.77) |
| Tan T (11) | III | 172 | III-IVB (UICC, 1997) | 86 | 86 | IC: gemcitabine 1,000 mg/m^2^ day  1, day 8; carboplatin AUC = 2.  5 day 1, day 8; paclitaxel 70 mg/  m^2^ day 1, day 8; Q3W for 3  cycles  CC: cisplatin 40 mg/m^2^ day 1;  Q1W for 8 cycles | CC: cisplatin 40  mg/m^2^ day 1;  Q1W for 8 cycles | Y | T N+ 69.96 Gy; N- 60Gy | 1.05  (0.50–2.19) | 0.77  (0.44–1.35) |
| Li WF (8) | III | 480 | III–IVB (AJCC-7) | 241 | 239 | IC: docetaxel 60 mg/m^2^ day 1;  cisplatin 60 mg/m^2^ day 1;  fluorouracil 600 mg/m^2^ days  1–5; Q3W for 3 cycles  CC: cisplatin 100 mg/m^2^ day 1;  Q3W for 3 cycles | CC: cisplatin  100 mg/m^2^ day 1;  Q3W for 3 cycles | Y | T N+ ≥66 Gy; N-≥50 Gy | 0.65  (0.43–0.98) | 0.67  (0.48–0.94) |
| Yang Q (13) | III | 476 | III–IVB, except T3N0–N1 (AJCC-6) | 238 | 238 | IC: cisplatin 80 mg/m^2^ day 1;  fluorouracil 800 mg/m^2^ days  1–5; Q3W for 2 cycles  CC: cisplatin 80 mg/m^2^ day 1;  Q3W for 3 cycles | CC: cisplatin 80  mg/m^2^ day 1;  Q3W for 3 cycles | Y | T 64-72 Gy; N- 48-50 Gy; N+ 60-66 Gy | 0.69  (0.49–0.98) | 0.66  (0.48–0.89) |
| Frikha M (15) | III | 81 | T2b, T3,  T4 and/or N1-N3, M0 (AJCC-6) | 40 | 41 | IC: docetaxel 75 mg/m^2^ day 1; cisplatin 75 mg/m^2^ day 1;  fluorouracil 800 mg/m^2^ days  1–5; Q3W for 2 cycles  CC: cisplatin 80 mg/m^2^ day 1;  Q3W for 3 cycles | CC: cisplatin 40  mg/m^2^ day 1;  Q1W for 7 cycles | Y | T 70 Gy | 0.40  (0.15–1.04) | 0.44  (0.20–0.97) |
| Hong RL (16) | III | 479 | IVA–IVB (AJCC-5) | 239 | 240 | IC: mitomycin 8 mg/m^2^ day 1;  epirubicin 60 mg/m^2^ day 1;  cisplatin 60 mg/m^2^ day 1;  fluorouracil 450 mg/m^2^ day 8;  leucovorin 30 mg/m^2^ day 8  CC: cisplatin 30 mg/m^2^ day 1;  Q1W for 8 cycle | CC: cisplatin 30  mg/m^2^ day 1;  Q1W for 8 cycle | Y | T ≥70 Gy; N+ 66–70 Gy | 0.92  (0.67–1.27) | 0.74  (0.57–0.97) |
| Zhang Y (7) | III | 480 | III–IVB (AJCC-7) | 242 | 238 | IC: gemcitabine 1,000 mg/m^2^ day  1, day 8; cisplatin 80 mg/m^2^ day  1; Q3W for 3 cycles  CC: cisplatin 100 mg/m^2^ day 1;  Q3W for 3 cycles | CC: cisplatin  100 mg/m^2^ day 1;  Q3W for 3 cycles | Y | T 66-70 Gy | 0.43  (0.24–0.77) | 0.51  (0.34–0.77) |
| Lee AWM (6) | III | 326 | III–IVB (AJCC-6) | 161 | 165 | IC: cisplatin 100 mg/m^2^ day  1, fluorouracil 1000 mg/m^2^ days  1–5; Q3W for 3 cycles  CC: cisplatin 100 mg/m^2^ day 1;  Q3W for 3 cycles | IC: cisplatin 100 mg/m^2^ day  1, capecitabine 2000 mg/m^2^ Q2W for 3 cycles  CC: cisplatin 100 mg/m^2^ day 1;  Q3W for 3 cycles | Y | T N+ ≥70 Gy (66 Gy for T1-T2a tumors); N- ≥50 Gy | 0.78  (0.41–1.41) | 0.7 (0.4–1.23) |
| Lee AWM (6) | III | 321 | III–IVB (AJCC-6) | 161 | 160 | IC: cisplatin 100 mg/m^2^ day 1, fluorouracil 1000 mg/m^2^ days  1–5; Q3W for 3 cycles  CC: cisplatin 100 mg/m^2^ day 1;  Q3W for 3 cycles | CC: cisplatin 100 mg/m^2^ day 1;  Q3W for 3 cycles  AC: cisplatin 80 mg/m^2^ day 1;  fluorouracil 1,000 mg/m^2^ days  1–4; Q4W for 3 cycles | Y | T N+ ≥70 Gy (66 Gy for T1-T2a tumors); N- ≥50 Gy | 0.59 (0.33–1.06) | 0.63 (0.37–1.05) |
| Lee AWM (6) | III | 365 | III–IVB (AJCC-6) | 165 | 160 | IC: cisplatin 100 mg/m^2^ day 1,  capecitabine 2000 mg/m^2^ Q2W for 3 cycles  CC: cisplatin 100 mg/m^2^ day 1;  Q3W for 3 cycles | CC: cisplatin 100 mg/m2 day 1;  Q3W for 3 cycles  AC: cisplatin 80 mg/m^2^ day 1;  fluorouracil 1,000 mg/m^2^ days  1–4; Q4W for 3 cycles | Y | T N+ ≥70 Gy (66 Gy for T1-T2a tumours); N- ≥50 Gy | 0.46 (0.25–0.82) | 0.44 (0.26–0.74) |
| Chen L (21) | III | 508 | III–IVB except T3–T4N0 (AJCC-6) | 251 | 257 | CC: cisplatin 40 mg/m^2^ day 1;  Q3W for 7 cycles  AC: cisplatin 80 mg/m^2^ day 1;  fluorouracil 800 mg/m^2^ days  1–5; Q4W for 3 cycles | CC: cisplatin 40  mg/m^2^ day 1;  Q3W for 7 cycles | N | T≥66 Gy;  N- 50 Gy;  N+ 60-66 Gy | 0.83 (0.57–1.22) | 0.88 (0.64–1.22) |

^a^ We arbitrarily considered trials with >50% patients treated with IMRT as studies adopting significant use of IMRT in this network meta-analysis.

AJCC-5/6/7, 5^th^/6^th^/ 7^th^ edition of the American Joint Committee on Cancer Staging Manual; CC, concurrent chemotherapy; CCRT, concurrent chemoradiation; IC, induction chemotherapy; AC, adjuvant chemotherapy; AUC, area under the concentration–time curve; IMRT, intensity-modulated radiation therapy; Y, yes; N, no; RT, radiotherapy; T, tumour; N-, negative neck lymph nodes; N+, positive neck lymph nodes; HR, hazard ratio; Q1/2/3/4W, every 1/2/3/4 weeks; UICC, Union for International Cancer Control;

**Supplementary Table 2. Quality assessment of the randomized-controlled trials selected**

| **Trials** | **Jadad Score** | **Randomization** | **Double Blinding** | **Drop-outs or Withdraws** | **Allocation Concealment** |
| --- | --- | --- | --- | --- | --- |
| Hui, EP (9) | 5 | 2 | 0 | 1 | 2 |
| Fountzilas, G (10) | 5 | 2 | 0 | 1 | 2 |
| Tan, T (11) | 5 | 2 | 0 | 1 | 2 |
| Li, WF (8) | 5 | 2 | 0 | 1 | 2 |
| Yang, Q (13) | 5 | 2 | 0 | 1 | 2 |
| Frikha, M (15) | 5 | 2 | 0 | 1 | 2 |
| Hong, RL (16) | 5 | 2 | 0 | 1 | 2 |
| Zhang, Y (7) | 5 | 2 | 0 | 1 | 2 |
| Lee, AWM ^a^ (6) | 5 | 2 | 0 | 1 | 2 |
| Chen L^b^ (21) | 5 | 2 | 0 | 1 | 2 |

^a^This study was used in both standard network meta-analysis and sensitivity analysis.

^b^This study was used in sensitivity analysis.

| **Study^a^** | **IC Regimen** | **Sample Size in IC-CRT, n** | **Grade 3 or Above Acute Adverse Events** | | | | | | | | | | | | | |
| --- | --- | --- | --- | --- | --- | --- | --- | --- | --- | --- | --- | --- | --- | --- | --- | --- |
|  |  |  | Hematologic, n (%) | | | | | Non-hematologic, n (%) | | | | | | | | |
|  |  |  | Leukopenia | Neutropenia | Febrile neutropenia | Thrombocytopenia | Anemia | Vomit, nausea, anorexia | Diarrhea | Mucositis, dysphagia, odynophagia | Fatigue | Allergic reaction | Hepatotoxicity | Nephrotoxicity | Weight loss | Alopecia |
| Hui EP (9) | DC | 34 | NR | 33 (97.1) | 4 (11.8) | 0 (0) | 0 (0) | 3 (8.8) | NR | 0 (0) | 2 (5.9) | NR | NR | NR | NR | NR |
| Fountzilas G (10) | PET | 72 | 0 (0) | 6 (8.3) | 0 (0) | 0 (0) | 1 (1.4) | 4 (5.6) | 0 (0) | 0 (0) | 1 (1.4) | 1 (1.4) | 0 (0) | 0 (0) | 0 (0) | 36 (50) |
| Tan T (11) | GCP | 86 | 16 (18.6) | 50 (58.1) | NR | 0 (0) | 1 (1.2) | 0 (0) | NR | NR | 0 (0) | 1 (1.2) | 2 (2.4) | NR | NR | 0 (0) |
| Yang Q (13) | PF | 238 | 12 (5.0) | 35 (14.7) | NR | 0 (0) | 1 (0.4) | 10 (4.2) | 1 (0.4) | 3 (1.3) | NR | 1 (0.4) | 2 (0.9) | 0 (0) | 0 (0) | NR |
| Frikha M (15) | TPF | 40 | NR | 11 (27.5) | 3 (7.5) | NR | NR | NR | NR | 5 (12.5) | 4 (10) | NR | NR | NR | NR | 6 (15) |
| Hong RL (16) | MEPFL | 239 | 139 (58.2) | NR | 10 (4.2) | 66 (27.6) | 16 (6.7) | 42 (17.6) | NR | 3 (1.3) | NR | NR | 4 (1.7) | 0 (0) | NR | NR |
| Zhang Y (7) | GP | 242 | 26 (10.7) | 49 (20.2) | 0 (0) | 13 (5.4) | 4 (1.7) | 48 (19.8) | 1 (0.4) | 2 (0.8) | NR | 1 (0.4) | 5 (2.1) | 3 (1.2) | 0 (0) | NR |

**Supplementary Table 3. Major acute adverse events during induction chemotherapy phase of each trial**

^a^ Studies from Li (8) and Lee (6) were excluded because the toxicities during the IC phase were not reported.

DC, docetaxel + cisplatin; PET, cisplatin + epirubicin + paclitaxel; GCP, gemcitabine + carboplatin + paclitaxel; IC, induction chemotherapy; TPF, docetaxel + cisplatin + fluorouracil; MEPFL, mitomycin + epirubicin + cisplatin + fluorouracil + leucovorin; PF, cisplatin + fluorouracil; GP, gemcitabine + cisplatin; NR, not reported.

**Supplementary Table 4. Major acute adverse events during concurrent chemoradiotherapy phase of each trial**

| **Study^a^** | **IC Regimen** | **Sample Size in IC-CRT, n** | **Grade 3 or Above Acute Adverse Events** | | | | | | | | | | | | | | |
| --- | --- | --- | --- | --- | --- | --- | --- | --- | --- | --- | --- | --- | --- | --- | --- | --- | --- |
|  |  |  | Hematologic, n (%) | | | | | Non-hematologic, n (%) | | | | | | | | | |
|  |  |  | Leukopenia | Neutropenia | Febrile neutropenia | Thrombocytopenia | Anemia | Vomit, nausea, anorexia | Diarrhea | Mucositis, dysphagia, odynophagia | Fatigue | Allergic reaction | Hepatotoxicity | Nephrotoxicity | Dermatitis | Weight loss | Dry mouth |
| Hui EP (9) | DC | 34 | NR | 9 (26.5) | 1 (2.9) | 3 (8.8) | 3 (8.8) | 3 (8.8) | NR | 8 (23.5) | 5 (14.7) | NR | NR | NR | NR | NR | NR |
| Fountzilas G (10) | PET | 72 | 16 (22.2) | 4 (5.3) | 0 (0) | 10 (13.9) | 3 (4.2) | 13 (18.0) | 2 (2.8) | 46 (63.9) | 0 (0) | 0 (0) | NR | 0 (0) | 4 (5.3) | 17 (23.6) | 6 (8.3) |
| Tan T (11) | GCP | 86 | 45 (52.3) | 21 (24.4) | NR | 12 (14.0) | 2 (2.3) | 2 (2.3) | NR | 26 (30.2) | 12 (14.0) | 1 (1.2) | 1 (1.2) | NR | 5 (5.8) | NR | 0 (0) |
| Li WF (8) | TPF | 241 | 98 (40.7) | 101 (41.9) | 7 (2.9) | 6 (2.5) | 4 (1.7) | 106 (44.0) | NR | 103 (42.7) | NR | 2 (0.8) | 7 （2.9） | NR | 9 (3.7) | NR | 13 (5.4) |
| Yang Q (13) | PF | 238 | 45 (18.9) | 24 (10.1) | NR | 4 (1.7) | 23 (9.7) | 25 (10.5) | 0 (0) | 16 (6.7) | NR | 0 (0) | 0 (0) | 1 (0.4) | 12 (5.0) | 3 (1.3) | 5 (2.1) |
| Hong RL (16) | MEPFL | 239 | 70 (29.3) | NR | 3 (1.3) | 78 (32.6) | 23 (9.6) | 17 (7.1) | NR | 82 (34.3) | NR | NR | 6 (2.5) | 0 (0) | 16 (6.7) | NR | NR |
| Zhang Y (7) | GP | 242 | 63 (26.0) | 67 (27.7) | 1 (2.9) | 27 (11.2) | 23 (9.5) | 109 (45.0) | 6 (2.5) | 69 (28.5) | NR | 0 (0) | 6 (2.5) | 6 (2.5) | 5 (2.1) | 5 (2.1) | 12 (5.0) |
| Lee AWM ^b^ (6) | PF | 161 | NR | (4) | NR | (2) | (8) | (2) | (0.4) | (37) | NR | NR | NR | (3) | (4) | (2) | NR |
| Lee AWM ^b^ (6) | PX | 165 | NR | (6) | NR | (4) | (8) | (2) | (1) | (33) | NR | NR | NR | (3) | (3) | (2) | NR |

^a^ The study from Frikha (15) was excluded because the toxicities during the CCRT phase were not reported.

^b^ This study reported only the cumulative rate (%) of major toxicity without the raw number of events.

DC, docetaxel + cisplatin; PET, cisplatin + epirubicin + paclitaxel; GCP, gemcitabine + carboplatin + paclitaxel; IC, induction chemotherapy; TPF, docetaxel + cisplatin + fluorouracil; MEPFL, mitomycin + epirubicin + cisplatin + fluorouracil + leucovorin; PF, cisplatin + fluorouracil; PX, cisplatin + capecitabine; GP, gemcitabine + cisplatin; NR, not reported.
